# Investigation of Epstein-Barr Virus and Parvovirus B19 DNA in Allogeneic Stem Cell Transplant Patients

**DOI:** 10.4274/tjh.2012.0042

**Published:** 2014-06-10

**Authors:** Altay Atalay, Selma Gökahmetoğlu, Süleyman Durmaz, İdris Kandemir, Derya Sağlam, Leylagül Kaynar, Bülent Eser, Mustafa Çetin, Hüseyin Kılıç

**Affiliations:** 1 Erciyes University Faculty of Medicine, Department of Clinical Microbiology, Kayseri, Turkey; 2 Erciyes University Faculty of Medicine, Department of Hematology, Kayseri, Turkey

**Keywords:** Epstein-Barr virus, Parvovirus B19, allogeneic stem cell transplantation, Real-time PCR

## Abstract

**Objective:** We aimed to investigate posttransplant Epstein-Barr virus (EBV) and parvovirus B19 DNA in allogeneic stem cell transplant patients between 2009 and 2010.

**Materials and Methods:** Forty-five adult patients in whom allogeneic stem cell transplantation was performed between April 2009 and November 2010 in the Erciyes University Faculty of Medicine, Department of Internal Medicine, Division of Hematology and Oncology, were included in the study. EBV and parvovirus B19 DNA positivity was investigated by using real-time polymerase chain reaction technique in 135 plasma samples obtained after transplantation at between 1 and 6 months. Pretransplant serological markers of EBV and parvovirus B19 were provided from patient files.

**Results:** In 32 (71.1%) of the patients, EBV antibodies in the pretransplantation period were as follows: anti-EBNA-1 IgG (+), VCA IgM (-), and VCA IgG (+). In 2 patients (4.45%), these antibodies were as follows: anti-EBNA-1 IgG (+), VCA IgM (-), and VCA IgG (-). In 1 patient (2.2%), they were as follows: anti-EBNA-1 IgG (-), VCA IgM (-), and VCA IgG (+). EBV serological markers were negative in 2 (2.2%) out of 45 patients before transplantation. There was low DNA positivity (<600 copies/mL) in 4 patients (8.9%), and VCA IgM was negative and VCA IgG was positive in these same 4 patients. In spite of low viral load, there were no symptoms related to EBV, and posttransplant lymphoproliferative disorder (PTLD) did not occur. While in 44 (99.7%) of 45 patients parvovirus B19 IgM was negative and IgG was positive, parvovirus B19 IgM was positive and IgG was negative in 1 (2.3%) patient. Parvovirus B19 DNA was not identified in any of the samples obtained from these 45 patients.

**Conclusion:** In this study, EBV and parvovirus B19 DNA were investigated in allogeneic stem cell transplant patients. None of the patients developed PTLD and parvovirus B19 DNA positivity was not detected. However, this issue needs to be further evaluated in prospective, multicenter studies with larger series of patients.

## Abstract (tr)

**Amaç:** Bu çalışmada, 2009-2010 yılları arasında allojenik kök hücre transplantasyonu (AKHT) yapılan hastalarda transplantasyon sonrası EBV ve parvovirus B19 DNA araştırılması amaçlandı.

**Gereç ve Yöntemler:** Bu çalışmaya Erciyes Üniversitesi Tıp Fakültesi İç Hastalıkları Anabilim Dalı Hematoloji-Onkoloji Bilim Dalı’nda Nisan 2009-Kasım 2010 tarihleri arasında AKHT yapılmış 45 erişkin hasta dahil edildi. Hastaların transplantasyon sonrası 1-6. aylar arasında alınan toplam 135 plazma örneğinde EBV ve parvovirus B19 DNA varlığı gerçek zamanlı PCR yöntemi ile araştırıldı. Hastalara ait transplantasyon öncesi EBV ve parvovirus B19 serolojik göstergeleri hasta dosyalarından temin edildi.

**Bulgular:** AKHT öncesi serolojik göstergelerde, 45 hastanın 32’sinde (%71,1) EBNA-1 IgG (+), VCA IgM (-) ve VCA IgG (+) idi. İki hastada (%4,45) EBNA-1 IgG (+), VCA IgM (-) ve VCA IgG (-), bir hastada (%2,2) EBNA-1 IgG (-), VCA IgM (-) ve VCA IgG (+) ve 2 (%4,45) hastada tüm serolojik göstergeler negatifti. Transplantasyon sonrası düşük EBV DNA pozitifliği (<600 kopya/mL) 4 (%8,9) hastada saptandı, bu hastaların hepsinde VCA IgM negatif, VCA IgG pozitif bulundu. Düşük viral yüke rağmen bu hastalarda EBV ilişkili semptom görülmemiş ve PTLD gelişmemiştir. Kırk beş hastanın 44’ünde (%97,7) parvovirus B19 IgM negatif, IgG pozitif iken sadece bir hastada (%2,3) parvovirus B19 IgM pozitif, IgG negatifti. Kırk beş hastadan elde edilen örneklerin hiçbirinde parvovirus B19 DNA saptanmadı.

**Sonuç:** Bu çalışmada AKHT hastalarında EBV ve parvovirus B19 DNA araştırıldı. Hastaların hiçbirinde PTLD gelişmezken parvovirus B19 DNA pozitifliği de saptanmadı. Ancak bu konunun aydınlatılmasında daha geniş hasta serileri ile yapılan, prospektif, çok merkezli ileri çalışmalara ihtiyaç vardır.

## INTRODUCTION

Allogeneic stem cell transplantation (ASCT) has been applied as a treatment option in an ever-increasing manner in various malignancies and hematological disorders for 40 years [1]. Posttreatment infections and graft-versus-host disease are the most common problems in ASCT [2]. Cytomegalovirus (CMV) is still the most important virus that infects hematopoietic stem cell and solid organ transplant recipients. However, the list of viruses that infect these patients and cause severe morbidity and mortality gets longer each day [3].

The Epstein-Barr virus (EBV) is a member of the family Herpesviridae. EBV infects almost all of the adult population in the world and stays persistent throughout life, as do all the other herpes viruses. Bone marrow transplant recipients carry a 4- to 7- fold increased risk of cancer compared to the normal population. Severe immune deficiency is observed within the first year after transplantation. Posttransplant lymphoproliferative disorder (PTLD) mostly appears in this period, and especially in the first 5 months. The mean PTLD incidence is 1% in allogeneic SCT recipients [4,5]. Human parvovirus (PV) B19 is the smallest DNA virus known so far, a nonenveloped microorganism of 18-26 nm in size [6]. Chronic PV B19 infections are described in many patients following stem cell transplantation. The infections encountered during chemotherapy in this patient group may mimic leukopenic relapses or therapy-induced cytopenias, and thus may cause misdiagnoses, unnecessary blood transfusions, and premature abortion of treatment [7,8]. Therefore, rapid diagnosis and treatment of PV B19 infections is very important in this patient group. 

The aim of this study was to investigate the occurrence of EBV-DNAemia after ASCT and its duration and magnitude, and to correlate these results with the appearance of EBV-driven PTLD. At the same time, another aim was to investigate the occurrence, duration, and magnitude of PV B19-DNAemia after ASCT.

## MATERIALS AND METHODS

Forty-five adult patients who underwent ASCT at the Erciyes University Medical Faculty, Department of Internal Medicine, Division of Hematology and Oncology, between April 2009 and November 2010 were included in the study. The presence of EBV and PV B19 DNA were investigated in a total of 135 plasma samples via real-time polymerase chain reaction (PCR) in the posttransplantation period (3 samples from each patient for EBV between the first and sixth months; 3 samples for each patient for PV B19 in the first, second, and third months). Two milliliters of blood samples with ethylenediaminetetraacetic acid was taken from all patients for the investigations of EBV and PV B19 DNAs. Plasma was separated following centrifugation and kept at -70 °C until analyses. DNA extraction from blood plasma was performed by the recommendations of the manufacturer using the EZ1 advanced virus kit (QIAGEN, Germany). DNAs were analyzed via real-time PCR using the EBV RG PCR kit (QIAGEN) for EBV and PV B19 RG PCR kit (QIAGEN) for PV B19. Extracted DNA (10 µL) was added to the plaques containing 15 µL of reaction mixture. An internal control of approximately 0.25 µL was added to the reaction mixture. PCR was conducted for 10 min at 95 °C, and then 45 cycles were repeated as follows: 1 cycle was composed of 15 s at 95 °C, 30 s at 55 °C, and 20 s at 72 °C. Amplification was performed in a Rotor-Gene 6000 instrument (Corbett Research, Australia). The quantitation range of the real-time PCR test for EBV was 600-600.000 copies/mL, and the analytic sensitivity was 157 copies/mL. For PV B19, the quantitation range was 1500-15.000.000 copies/mL and the analytic sensitivity was 30 copies/mL. The serological EBV and PV B19 indicators of patients were obtained from the patient files. The ethics committee approved this study.

**Statistical**

Statistical analysis was performed using SPSS 13.0. Categorical variables are presented as numbers and percentages, and continuous variables are presented as mean ± SD or median.

## RESULTS

The demographical characteristics of patients included in the study are shown in Table 1. HLA-matching status for ASCT was full-match in 35 patients and all of the allogeneic transplant donors were related. According to the pre-ASCT serological indicators of the patient files, 32 of 45 patients (71.1%) were EBNA-1 IgG (+), VCA IgM (-), and VCA IgG (+). Two patients (4.45%) were EBNA-1 IgG (+), VCA IgM (-), and VCA IgG (-); 1 patient (2.2%) was EBNA-1 IgG (-), VCA IgM (-), and VCA IgG (+); and 2 patients (4.45%) were serologically negative for all indicators. EBNA-1 IgG results could not be obtained for 8 (17.8%) patients. Six of these 8 patients (75%) were VCA IgM (-) and VCA IgG (+), whereas 2 (25%) were VCA IgM (-) and VCA IgG (-). EBV-specific antibody EA was negative in 34 patients (75.5%) and positive in 1 patient (2.2%). The EA-positive patient was VCA IgM (-), VCA IgG (+), and EBNA-1 IgG (+). EA results could not be obtained for 10 patients (22.3%). EBV DNA was positive (<600 copies/mL) in 4 patients (8.9%), and they were all VCA IgM (-) and VCA IgG (+). EBV DNA positivity of these patients belonged to the posttransplantation third month in 2 patients, fifth month in 1 patient, and sixth month in 1 patient (Table 2). No EBV-related symptom or PTLD was observed in these patients despite the low viral load. We wanted to follow the results of all 4 patients that had low EBV viral load, but 1 of them died and 1 went to another hospital for treatment. We were able to check the results of the other 2 patients, and EBV DNA was found to be negative. 

In 44 (97.7%) of 45 patients, PV B19 IgM was negative and IgG was positive, whereas in only 1 patient (2.3%), PV B19 IgM was positive and IgG was negative. No PV B19 DNA was observed in the samples obtained from these 45 patients. EBV and PV B19 serology of the donors and patients included in the study are shown in Table 3.

## DISCUSSION

Determining a patient’s risk factors concerning posttransplantation viral infection development may be possible by detecting the virological situations of both the donor and the recipient just before transplantation. However, this detection alone is not enough; the recipients should be followed regularly after the transplantation and tested in respect to possible viral infections [9]. The viral infections of the posttransplantation period may vary according to the type of the transplant. In patients with stem cell or bone marrow transplantation, polyoma, herpes simplex (HSV), and respiratory and enteric viral infections are common in the first month following the transplantation. In solid organ transplantation recipients, HSV infections are common in the first month following transplantation. In both groups, CMV, EBV, and varicella zoster virus infections may be seen after the second month. Periodic follow-up for viruses other than CMV is suggested in the posttransplantation period. However, no standardization has been provided yet [10]. 

The serological status of EBV in transplant recipients should be certain before the transplantation since seronegativity is a risk factor. Serology is limited since the severe immunosuppression of transplant recipients after the transplantation procedure inhibits the production of sufficient antibodies. Follow-up of the viral load by nucleic acid amplification tests is the preferred method [11]. Sometimes EBV may infect T/NK cells and cause persistent EBV infection. As a result, a high viral load and EBV-related T/NK cell lymphoproliferative disease follow. Therefore, the quantitation of EBV viral load is important, and the most common method used for this purpose is real-time PCR [12]. PCR-based tests are useful not only in prediction and diagnosis of PTLD, but also in monitoring the response to the treatment [13]. In ASCT patients with PTLD, EBV DNA load is generally higher than that of ASCT patients without PTLD. However, no consensus has been reached concerning which EBV DNA load threshold values cause high risk for EBV-related disease or PTLD development [14]. On the other hand, patients with immunodeficiency may not manifest EBV-related symptoms despite the detectable EBV DNA in their serum or plasma samples [15]. In our study, EBV-related symptoms or PTLD was not present in any of the patients who were detected to be positive for EBV DNA via real-time PCR. Agbalika et al. [16] mentioned that EBV EA-IgG presence in pre-ASCT patients increased the incidence of early PTLD development in the first year following transplantation. In this study, EBV DNA positivity was detected in 4 posttransplantation patients, and the positivity times were as follows: third month in 2 patients, fifth month in 1 patient, and sixth month in 1 patient. EA-IgG was negative in 34 (75.5%) patients and positive in 1 (2.2%) patient. EA-IgG results could not be obtained in 10 cases (22.3%). No EA-IgG positivity was detected before transplantation in any of the 4 patients with EBV DNA positivity. 

PV B19 infections may be met in immunosuppression situations such as congenital or acquired immune deficiency syndrome, organ or bone marrow transplantation, lymphoproliferative disorders, malignancies, and chemotherapy [17]. In immunosuppressed patients, the clinical findings of PV B19 infection are severe, and viral eradication is late or inadequate. That may cause an aplastic crisis in patients with chronic hemolytic anemia [18]. 

Plentz et al. [19] detected PV B19 DNA in 21 (1%) of 2123 tested blood samples that were going to be transplanted to patients with hematological cancer [18]. In the posttransplantation period of patients with ASCT, primary or recurrent PV B19 incidence is found to be 1%-2% when sensitive diagnostic methods are used [8,19]. As PV B19 cannot proliferate in continuous cell cultures and serological diagnosis is not reliable in immunosuppressed patients, viral particle- or viral DNA-detecting methods such as PCR and nucleic acid hybridization are needed for diagnosis [20,21]. Manaresi et al. [22] demonstrated that PV B19 DNA could be detected by PCR even if the samples were IgM- and IgG-negative. They also noted the importance of real-time PCR in laboratory diagnosis of PV B19 infections by pointing out that real-time PCR is 5-fold faster than PCR-ELISA and has a high specificity/sensitivity and low contamination risk, and quantitation is possible. Harder et al. [23] emphasized the usefulness of quantitation of viremia by real-time PCR in immunosuppressed, reinfected children for the planning of their treatment. In our study, no PV B19 DNA was detected in any of the posttransplantation patients, 44 of whom were detected to be PV IgM (-) and IgG (+), and 1 of whom was IgM (+) and IgG (-) before ASCT. 

As a conclusion, in this study investigating EBV and PV B19 DNAs in ASCT patients, no PTLD development or PV B19 DNA positivity was observed. However, further prospective, multicenter studies with wider patient series should be conducted in order to better clarify the subject. 

## CONFLICT OF INTEREST STATEMENT

The authors of this paper have no conflicts of interest, including specific financial interests, relationships, and/or affiliations relevant to the subject matter or materials included.

## Figures and Tables

**Table 1:**
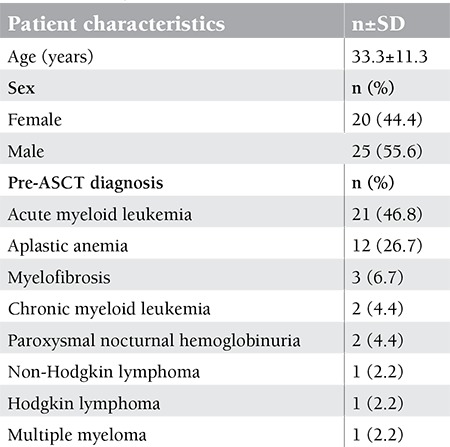
Demographic characteristics of patients.

**Table 2:**
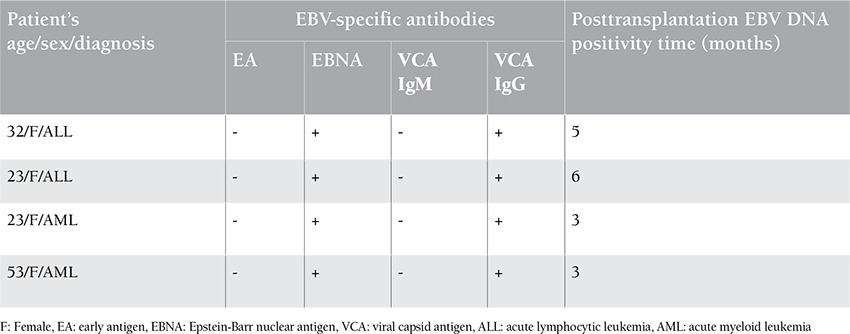
Demographic characteristics, serological indicators, and positivity times of patients with positive EBV DNA.

**Table 3:**
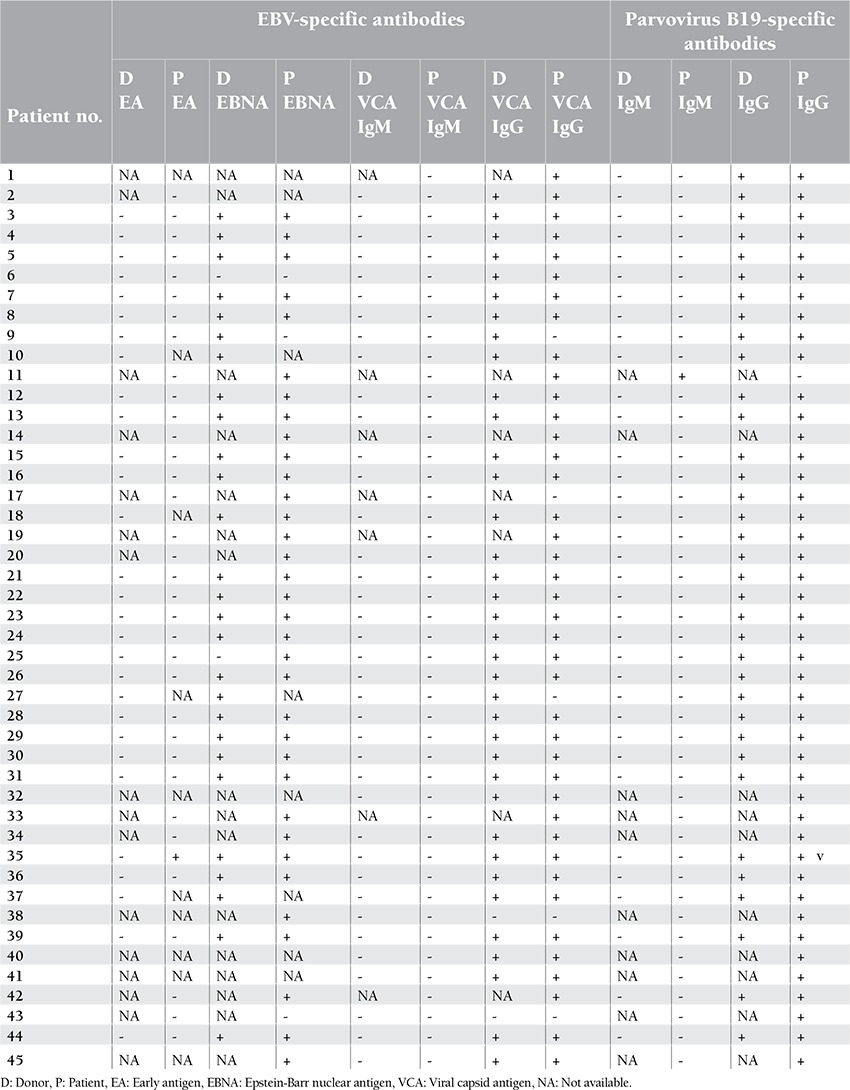
EBV and PV B19 serology of the donors and patients.
